# Sprint Conditioning of Junior Soccer Players: Effects of Training Intensity and Technique Supervision

**DOI:** 10.1371/journal.pone.0121827

**Published:** 2015-03-23

**Authors:** Thomas Haugen, Espen Tønnessen, Øyvind Øksenholt, Fredrik Lie Haugen, Gøran Paulsen, Eystein Enoksen, Stephen Seiler

**Affiliations:** 1 Norwegian Olympic Sports Program (Olympiatoppen), Sognsveien 228, 0840 Oslo, Norway; 2 Faculty of Health and Sport Sciences, University of Agder, Gimlemoen 25, 4630 Kristiansand, Norway; 3 Norwegian School of Sport Sciences, Sognsveien 220, 0806 Oslo, Norway; Norwegian University of Science and Technology, NORWAY

## Abstract

The aims of the present study were to compare the effects of 1) training at 90 and 100% sprint velocity and 2) supervised versus unsupervised sprint training on soccer-specific physical performance in junior soccer players. Young, male soccer players (17 ±1 yr, 71 ±10 kg, 180 ±6 cm) were randomly assigned to four different treatment conditions over a 7-week intervention period. A control group (CON, *n*=9) completed regular soccer training according to their teams’ original training plans. Three training groups performed a weekly repeated-sprint training session in addition to their regular soccer training sessions performed at A) 100% intensity without supervision (100UNSUP, *n*=13), B) 90% of maximal sprint velocity with supervision (90SUP, *n*=10) or C) 90% of maximal sprint velocity without supervision (90UNSUP, *n*=13). Repetitions x distance for the sprint-training sessions were 15x20 m for 100UNSUP and 30x20 m for 90SUP and 90UNSUP. Single-sprint performance (best time from 15x20 m sprints), repeated-sprint performance (mean time over 15x20 m sprints), countermovement jump and Yo-Yo Intermittent Recovery Level 1 (Yo-Yo IR1) were assessed during pre-training and post-training tests. No significant differences in performance outcomes were observed across groups. 90SUP improved Yo-Yo IR1 by a moderate margin compared to controls, while all other effect magnitudes were trivial or small. In conclusion, neither weekly sprint training at 90 or 100% velocity, nor supervised sprint training enhanced soccer-specific physical performance in junior soccer players.

## Introduction

The importance of sprinting in professional soccer is well established and the need for speed is clear [1–4]. According to track-and-field statistics [5], trends over time from large retrospective data collections in soccer players [3,4] and the experience of practitioners [6], sprint performance is resistant to training enhancement. Athletes can spend years training to improve a few hundredths of a second over short distances [5]. Numerous intervention studies have been performed over the years in order to enhance soccer-specific sprinting. A recent review reveals that sprinting under assisted, resisted and normal conditions, maximal and explosive strength training, plyometric training and high-intensity running have been investigated in different combinations, but no specific training method has so far emerged as superior [1]. Time efficiency is an important constraining aspect of team-sport conditioning and extensive off-field interventions will most likely be rejected by team coaches, independent of intervention efficacy [1].

The term ‘direct supervision’ refers to training situations in which a supervisor or training expert is present at all times [7,8]. The supervisor oversees training activities as they occur and provides direction, instruction, feedback and assistance. The importance of guidance and feedback during practice is well known in motor skill learning and performance enhancements may happen immediately in such settings [9]. Mazzetti et al. [7] and Coutts et al. [8] concluded that the presence of a training expert was beneficial for maximal strength development over time. To the authors’ knowledge, the effect of supervised sprint-training sessions in soccer players has not been investigated. According to motor skill learning theories, errors increase with the speed of the movement [9]. Technical training of typically rapid or ballistic movements should be interfered with by using specific drills, large amount of repetitions and an intensity where the athletes are able to control the movements (proper execution not interfered by fatigue). If the movement is slowed down slightly, the same generalized motor program can be used as in the normal-speed version [9]. In contrast, the vast majority of studies involving sprint-training interventions for soccer players make no recommendations other than that sprint velocity should be maximal throughout [1]. Available evidence in endurance and strength training demonstrates that high, but sub-maximal intensity loading effectively stimulates adaptation through the interaction between high intensity and larger accumulated work that can be achieved before the onset of fatigue, compared to maximal efforts [10,11]. This makes it tempting to speculate similar effects on sprinting. Anecdotal evidence in support of this is observed in the sprint-training philosophy developed by the athletic sprint pioneer coach Carlo Vittori in the mid-1970s [12]. His successful athletes performed repeated-sprint training sessions with an intensity as low as 90% of maximal sprint speed during initial pre-season conditioning in order to improve sprint endurance (later termed repeated-sprint performance). Inspection of training diaries reveals that internationally-competing sprinters perform sprint training with varying intensity through all parts of the season (unpublished material, Norwegian Olympic Federation). However, the lowest effective sprinting intensity for stimulating adaptation is so far not established in the research literature. Recently, Haugen et al. [13] observed that repeated 20-m sprints at 90% intensity did not enhance sprint performance during a soccer season. It was suggested that such training should be performed at other times of the season to avoid training-related constraints due to the high volume of overall soccer conditioning. The aims of the present study were therefore to compare the effects of 1) training at 90 and 100% sprint velocity and 2) supervised versus unsupervised sprint training at 90% sprint velocity on soccer-specific physical performance capacities in junior soccer players’ early in pre-season.

## Materials and Methods

### Ethics statement

This study was conducted in accordance with the declaration of Helsinki. All participants provided written, voluntary informed consent before participation. Written parental consent was also provided for participants < 18 yr old. The human subjects review committee of the Faculty for Health and Sport, University of Agder, approved the study.

### Experimental approach to the problem

In this randomized controlled trial, participants were randomly assigned to four different treatment conditions. A control group (CON) completed regular soccer training according to their teams’ original early pre-season training plans. Three training groups performed a weekly repeated-sprint training session in addition to their regular soccer training sessions, which was performed at A) 100% intensity without supervision (100UNSUP), B) 90% of maximal sprint speed with supervision (90SUP) or C) 90% of maximal sprint speed without supervision (90UNSUP). Based on sample size limitations and motor learning principles identified in the introduction, the present study was not performed with a factorial design (i.e. an additional “100SUP” group). The duration of the intervention period was 7 weeks. To evaluate the treatment conditions (independent variables), the following soccer-specific performance tests (primary dependent variables) were assessed prior to and after the intervention period: 15x20 m repeated-sprint, countermovement jump (CMJ) and Yo-Yo Intermittent Recovery 1 (Yo-Yo IR1). To investigate possible mechanistic influences regarding adaptations to sprint training, the following secondary dependent variables were assessed during the 15x20 m repeated-sprint pre- and post-training tests: Heart rate, blood lactate concentration, step length and step rate. Finally, sprint times for all training sessions were assessed for intensity control (90SUP and 90UNSUP) and to examine weekly changes in repeated-sprint performance (100UNSUP).

### Participants

Fifty-two male junior soccer players, aged 16–19 years, volunteered to participate. The athletes were playing in the highest junior division level for four different clubs (*n* = 6,13,16 and 17) in Norway. Each participant had a minimum two years of soccer-specific conditioning experience. During the intervention period, the participants were requested to refrain from performing any other off-field physical training regimes in terms of speed, strength and/or endurance. All participants were free of injuries prior to preliminary testing. None of the athletes had previous experience with specialized repeated-sprint training.

To eliminate the influence of varying overall soccer conditioning, the participants were initially distributed by club and then allocated to one of the four intervention conditions by a co-author not directly involved in testing or the training intervention. The 14 participants randomly assigned to each of the three training groups were required to complete at least six out of seven training sessions during the intervention period in addition to all performance tests in order to be included in further analyses. The 10 allocated CON participants were required to perform at least 80% of planned sessions and complete all pre- and post-training tests. We chose a slightly uneven distribution of subjects based on 1) the expectation of increased dropout risk generally observed in any intervention and 2) the expectation of lower variability of outcome in CON exposed to testing only and an unchanged training routine.

One participant each from CON, 100UNSUP and 90SUP dropped out due to illness during training or testing. Two participants from 90SUP and one from 90UNSUP dropped out due to injuries sustained outside the sprint-training intervention. A final player from 90SUP group dropped out due to Achilles tendon strain, possibly associated with the sprint intervention. Thus, 45 of 52 participants completed the study with the following sample sizes (club distribution in brackets): CON = 9 (0,3,3,3), 100UNSUP = 13 (0,4,5,4), 90UNSUP = 13 (1,3,5,4) and 90SUP = 10 (2,3,3,2). Physical and training characteristics of these participants are presented in Table 1.

**Table 1:** Physical and training characteristics at inclusion.

Group	n	Age (yr)	BM (kg)	Height (cm)	Weekly training sessions	Games per week (n)	Tot. vol. (h·wk-1)
CON	9	17 ±1	72 ±11	181 ±6	4.4 ±2.3	0.4 ±0.4	6.8 ±3.3
100UNSUP	13	17 ±1	66 ±9*	178 ±6	4.4 ±2.3	0.3 ±0.7	6.6 ±3.8
90UNSUP	13	17 ±1	72 ±6	183 ±5	4.5 ±2.4	0.4 ±1.0	7.0 ±3.5
90SUP	10	17 ±1	72 ±8	178 ±7	4.4 ±1.6	0.4 ±0.9	6.8 ±2.9

Values are mean ± SD. BM = Body mass, Tot. vol. = Total training volume. Training values are based on self-reported weekly averages during the intervention period. There were no significant differences among the groups for any of the variables, except for body mass (*100UNSUP < 90UNSUP, *p* = 0.04).

Regular soccer training sessions typically commenced with warm-up activities like short-passing and coordination exercises with the ball, followed by more intensive change-of-direction exercises with and without ball. The main part of the soccer practice consisted of small-sided and more full-sized team compositions, ranging from 3 vs. 3 to 7 vs. 11.

### Testing procedures

The pre- and post-training tests were conducted at the Norwegian Olympic Training Centre on two separate days, with two days in between. All participants completed the tests in the same order and at the same time of day. Regarding nutrition, hydration, sleep and physical activity, the athletes were instructed to prepare as they would for a regular soccer match, including no high-intensity training the last two days before testing. They were also instructed to use identical footwear and kit for each of the tests. Test day one consisted of CMJ and 15x20 m repeated- sprint testing. On test day two, the athletes completed the Yo-Yo IR1 test. Prior to testing on test-day 1, participants completed a 25 min standardized treadmill warm-up consisting of a 10-min general warm-up at 60–75% of age-predicted maximum heart rate, 3 sets of 4 exercise drills (high knees, back kick, sideway and backwards running) and finally 2–3 repetitions of 40-m runs with a progressive increase in speed. Prior to testing on test-day 2, participants warmed up with a 10-min easy jog at 60–75% of age-predicted heart rate followed by the initial 60–90 s of the Yo-Yo IR1 test.

#### CMJ test

Immediately after warm up, each athlete was weighed on a force platform for system calibration before performing three trials of CMJ (vertical jump) separated by 1 min recovery. The best result for each player was retained for analysis. To isolate leg extensor muscles and minimize technical elements, all jumps were performed with hands placed on the hips. The tests were performed on an AMTI force platform (OR6–5–1, Watertown, USA). Calculation of jump height is formerly described in Haugen et al. [3].

#### Sprint test

A 15x20 m repeated-sprint test with starts each 60 s was performed directly after the CMJ test. Distance and recovery were chosen in line with mean frequency and typical distance of all-out sprints reported from match analyses [14]. Procedures and equipment are formerly described in Haugen et al. [13]. Best 20-m time was used in order to determine maximal single-sprint capacity, while mean time for the 15 sprints was used to determine repeated-sprint performance. Heart rate was measured continuously during the test (Polar RS400, Kempele, Finland). A blood sample was acquired via finger stick to quantify the blood lactate concentration (BLa^-^) immediately after the last sprint (LactatePro LT-1710, Arkay KDK, Kyoto, Japan).

All sprint tests were captured by a video camera (Sony HDR-HC9E)) mounted on a tripod in line with the finish line and 3 m from the sprinter’s running lane. Video recordings were analysed in ProSuite, version 5.5 (Dartfish, Switzerland) to determine step count and derive average step length (SL). For precision, the digital ruler in the analyser window was used to interpolate the last step across the finish line. For example; if the 13^th^ and 14^th^ ground contact occurred 0.8 m in front of and 1.2 m beyond the finish line, respectively, the recorded number of steps was registered as 13.4. Mean SL was calculated by dividing the number of steps by the distance (in this case: 20 m·13.4^-1^ = 1.49 m). Mean step rate (SR) was calculated from mean velocity and mean SL. Prior to the present study, this measurement method was validated by rolling out thin paper at the finish line area in order to measure the distance between the visible spike shoe marks from competitive sprinters. The absolute difference across twenty sprint comparisons never exceeded 0.1 steps. Thus, the maximal margin of error for step counts over 20 m is 0.7–0.8% for athletes using 13–15 steps.

#### Yo-Yo IR1 test

The Yo-Yo IR1 test was performed indoors on artificial turf. Two test leaders supervised the tests. The athletes were divided in small groups that completed the test consecutively, such that each supervisor was responsible for ≤ 5 athletes during the test. Set-up and procedures were in line with the guidelines by Krustrup et al. [15], who have reported a test-retest CV < 5%. The test score is reported in total distance covered until exhaustion.

### Intervention program

The training intervention took place from the end of October to mid December, corresponding to early pre-season in the Norwegian soccer annual cycle. The sprint-training sessions were performed at the same time and day for each training group throughout the intervention period and no regular soccer training sessions were performed on the same day as the sprint training took place. Athletes in 100UNSUP performed 15x20 m maximal sprints with starts each 60 s once a week. Groups 90SUP and 90UNSUP performed one weekly training session consisting of a larger dose of 30x20 m sprints at 90% of maximal sprint velocity (based on the best 20-m sprint time obtained during the pre-training test) with starts each 60 s.

Two sprint-training experts, with extensive national-level coaching experience, supervised the 90SUP group during the intervention. Three key sprint-technical elements and corresponding verbal instructions were emphasized during the training sessions:

- Optimal upper-body angle relative to the ground during the initial steps in order to create higher horizontal propulsive forces through more effective utilization of hip and knee extensors [16,17]. The athletes were instructed to assume a start position with forward lean in the upper body and a lowered centre of gravity and to gradually become more upright throughout the acceleration.
- Minimize horizontal braking forces [16]: Athletes with apparently too high braking forces were encouraged to assume a more favourable configuration at the point of ground contact with the foot plant closer to the perpendicular line from the centre of mass. This can be achieved by hitting the ground with a bent knee (relevant during acceleration) or with the centre of mass at a large vertical distance above the ground (relevant during maximal sprinting).
- Produce a stiff rebound during ground contact in order to minimize degeneration of horizontal propulsive forces [18–20]: Identified “heal runners” were encouraged to pre- activate dorsiflexion muscles prior to foot plant and stiffen the ankle joint during ground contact, allowing them to utilize the elasticity in the plantar flexors for greater force development. These instructions were emphasized during the warm up drills.

After video analysis of the first training session, the two sprint-training experts prepared an individual capacity profile for all participants in the 90SUP group. Each athlete was presented with one technical task at a time, in accordance with general feedback principles [9]. Players with obvious technical limitations were provided with more verbal instructions than technically well-performing athletes.

In the absence of previously published studies, a 1:2 repetition ratio between 100% and 90% sprinting was chosen. Several measurements were assessed in order to compare the two repeated-sprint training sessions used. Firstly, session rated perceived exertion (RPE) was recorded for all athletes after the repeated sprints performed in pre-training testing and the first training session. Written and verbal instructions regarding its use were provided in advance [21]. Moreover, heart rate was measured continuously during the first training session for all athletes who ran at 90% sprint intensity, in addition to BLa^-^ immediately after their last sprint. These were compared to corresponding data assessed during pre-training tests. Mean SL and SR for the first sprint-training session were calculated by identical procedures as for the pre- and post-training tests. Finally, all training group athletes performed 3x20 m maximal sprints with starts each 60 s 48 hours after the first training session for a performance recovery check. The mean time for these three sprints was compared with corresponding sprints from the pre-training test.

**Fig 1:**
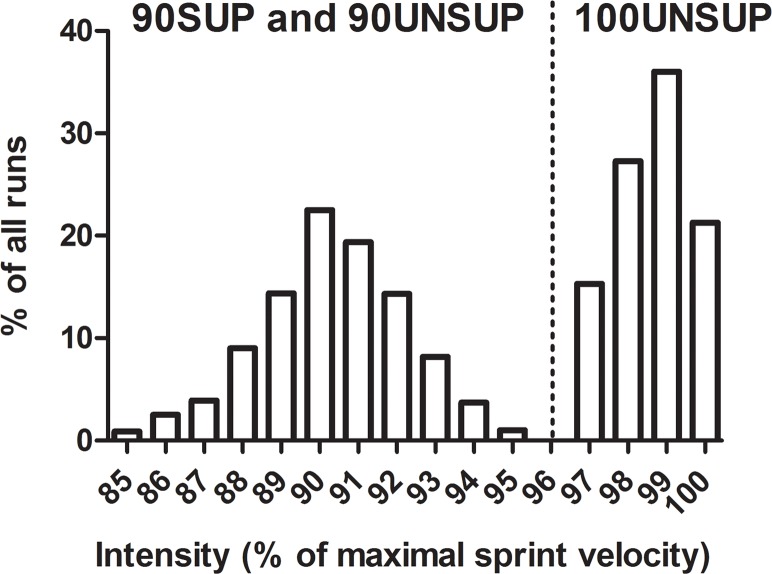
Intensity distribution for the sprint training groups during all training sessions. Best sprint from pre-training testing was set as reference (100%) for 90SUP and 90UNSUP, while best sprint within each training session was set as reference (100%) for 100UNSUP.

Electronic timing was continuously used to control running speed and adjust intensity according to each player’s “target time”. Target time for the 90SUP and 90UNSUP participants were derived from the best single-sprint time achieved during preliminary testing by multiplying mean velocity over the 20-m distance by 0.9. No feedback other than sprint time information (for intensity control purposes) was provided by a timekeeper for the 90UNSUP and 100UNSUP groups after each run. Fig. 1 shows intensity distribution for the two 90% groups (90SUP and 90UNSUP pooled together) during all training sessions. More than 90% of all sprints were completed with intensities between 87 and 93% of maximal sprint velocity. All sprints for 100UNSUP during the training sessions were completed with an intensity > 97% (mean ± SD: 98.2 ± 0.8%) when related to the best single-sprint within each training session. Thus, treatment conditions in 90SUP and 90UNSUP were strictly separated and did not overlap with 100UNSUP. For simplicity, we continue to use the terms “100UNSUP” or “maximal intensity”.

### Statistical analysis

All statistical analyses were carried out using SPSS 17.0 for Windows (SPSS Inc., Chicago, IL, USA). Level of significance was set to *p*<0.05. The General Linear Model with Repeated Measures followed by Bonferroni adjustment for multiple comparisons was used to examine repeated-sprint performance development (mean sprint time) for 100UNSUP across tests and training sessions. The same model was used for 90SUP and 90UNSUP (both groups pooled together) to compare effort-related variables in maximal and sub-maximal sprinting. Analysis of covariance (ANCOVA) adjusting for the pre-training test value and randomization stratification factor (club) was used to examine within-group and between-group mean changes. The differences were judged by using estimated marginal means (EMM). Bonferroni corrections were used to adjust *p*-values for multiple testing. Effect magnitudes were calculated and interpreted categorically according to the guidelines by Hopkins et al. [22]. The first 6 sprints from the pre-training test (for all included participants) were used to calculate typical variation for sprint time, SL and SR. Effect size of the within-group changes for mean sprint time were based on mean change and typical variation. The results are expressed as mean ±SD and 95% confidence intervals (95% CIs) were calculated for all measures.

## Results

Table 2 shows effort-related variables for the two repeated-sprint training sessions used in the present intervention. No differences in RPE were observed between the sessions. Mean sprint time for the 3x20-m sprints performed 48 hours after the first training session was not significantly different when compared to the corresponding pre-training sprint test. Sprinting at 90% velocity was accompanied with reduced HR _peak_ (17%; very large effect; *p*<0.001), BLa^-^ (55%; large effect; *p*<0.001) and SR (11%; very large effect; *p*<0.001) compared to maximal sprinting.

**Table 2:** Effort-related variables in maximal (100%) and sub-maximal (90%) sprinting.

Sprint session	15x20m (100% intensity)	30x20m (90% intensity)
Δ sprint time 48 h (s)	0.00 ±0.02	0.01 ±0.02
Session RPE	3.8 ±1.2	4.0 ± 1.1
HR _peak_ (beats· min^-1^)	170 ±10	141 ±10*
BLa^-^ (mmol·L^-1^)	4.4 ±1.8	2.0 ±0.7*
SL (m)	1.55 ±0.08	1.56 ±0.09
SR (steps·s^-1^)	4.36 ±0.18	3.87 ±0.22*

Δ sprint time 48 h = sprint time 48 hours after the first training session minus corresponding pre-training sprint test time (mean of first 3 sprints for each time point), RPE = rated perceived exertion, HR _peak_ = peak heart rate, BLa^-^ = blood lactate concentration, SL = step length, SR = step rate

* = significantly different from 100% sprinting (*p*<0.001).

**Fig 2:**
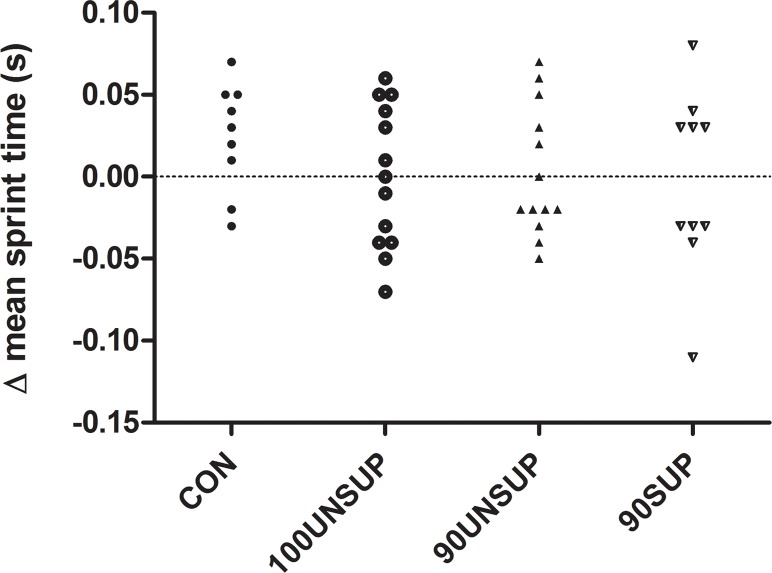
Individual changes in 15x20 m mean sprint time from pre- to post-training tests.

No significant within-group differences for the analyzed performance parameters were observed, except that 90SUP improved Yo-Yo IR1 performance from pre- to post-training (258 m; 17,3%; *p*<0.01). No significant between-group differences were observed (*p*<0.05). 90SUP improved Yo-Yo IR1 performance by a moderate margin compared to all other groups, while all other between-group differences were small or trivial (Table 3 and Fig. 2).

**Table 3:** Between-group changes (mean and 95% CIs) versus controls in physical performance from pre- to post-training.

Intervention group	Best sprint time (s)	Mean sprint time (s)	CMJ (cm)	Yo-Yo IR1 (m)
100UNSUP	-0.03 (-0.07 to 0.00)	-0.03 (-0.06 to 0.01)	1.0 (-0.6 to 2.6)	-34 (-272 to 205)
90UNSUP	-0.03 (-0.07 to 0.01)	-0.02 (-0.06 to 0.02)	0.4 (-1.3 to 2.1)	-1 (-120 to 117)
90SUP	-0.02 (-0.06 to 0.02)	-0.03 (-0.07 to 0.01)	1.8 (0.0 to 3.6)	131 (-108 to 369)

The differences vs. control group are assessed by estimated marginal mean. Minus (-) indicates lower values post-training

compared with the control group (assessed by estimated marginal means). CMJ = countermovement jump, Yo-Yo IR1 = Yo-Yo intermittent recovery level 1. No significant between-group differences were observed.

Achievement of best sprint performance was randomly distributed across the 15 sprints in all groups during both pre- and post-training tests. Typical variation for sprint time, SL and SR was 0.025 s (CV 1.0%), 0.028 m (CV 1.8%) and 0.08 strides·s^-1^ (CV 1.9%), respectively. In CON, a variation in mean sprint time of ±0.04 s was observed between the pre- and post-training tests. Corresponding variation for SL and SF was 0.06 m and 0.19 strides·s^-1^, respectively.

In 100UNSUP, significant differences from pre- to post-training tests were observed for BLa^-^ (1.5 mmol·L^-1^; 35.7%; *p*<0.001), SL (-0.04 m; 2.6%; *p* = 0.020) and SF (0.13 steps·s^-1^; 3.0%; *p* = 0.019). BLa^-^ increased significantly in 100UNSUP compared to CON from pre- to post-training (*p* = 0.008) (Table 4). No other within- or between-group differences were observed. The change in BLa^-^ within 100UNSUP was moderate while the other effect magnitudes between- or within-groups were trivial or small.

**Table 4:** Between group changes (mean and 95% CIs) versus controls for underlying performance variables between pre- and post-training.

Intervention group	Body mass (kg)	HR_peak_ (beats·min-1)	BLa- (mmol·L-1)	SL (m)	SR (steps·s-1)
100UNSUP	0.3 (-0.8 to 1.5)	5 (-1 to 12)	1.9 (0.7 to 3.2)*	0.00 (-0.07 to 0.06)	0.06 (-0.13 to 0.25)
90UNSUP	-0.3 (-1.4 to 0.8)	2 (-5 to 8)	1.1 (-0.1 to 2.3)	0.04 (-0.02 to 0.10)	-0.09 (-0.28 to 0.10)
90SUP	-0.3 (-1.5 to 0.9)	4 (-3 to 11)	1.5 (0.2 to 2.9)	0.03 (-0.03 to 0.10)	-0.04 (-0.24 to 0.17)

The differences vs. control group are assessed by estimated marginal mean. Minus (-) indicates lower values post-training compared with the control group (assessed by estimated marginal means). HR = heart rate, BLa^-^ = blood lactate concentration, SL = step length, SR = step rate

* = significantly different (Bonferroni adjusted) from CON (*p* = 0.01).

**Fig 3:**
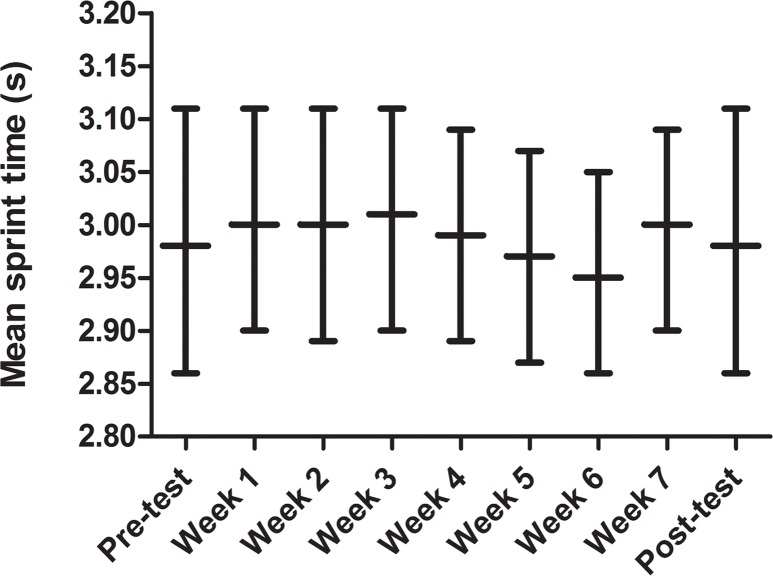
95% confidence intervals of mean sprint time for 100UNSUP during the intervention.

Fig. 3 shows the development of repeated-sprint performance (mean sprint time) for 100UNSUP during the intervention period, including pre- and post-training tests. Weekly changes in group mean values up to 0.05 s were observed.

## Discussion

In the present study, weekly repeated-sprint training sessions performed at maximal or with 90% intensity were not sufficient to improve soccer-specific physical performance in junior soccer players, when compared to a matched control group assumed to maintain a constant training pattern. Moreover, no differences in performance outcomes were observed between supervised and unsupervised sprint-training groups training at 90% maximal sprinting velocity. Apparently, the relative work loads elicited by the current intervention strategies were not sufficient to create appropriate adaptations during the early pre-season soccer period.

To the authors’ knowledge, this is the first study to compare the effects of sprint training at 90 vs. 100% sprint intensity or supervised vs. unsupervised sprint training. Our findings confirm the assumption that sprint performance is resistant to training enhancement, even among junior soccer players during the early pre-season period where total training load is reduced. Since treatment allocation was adjusted for club participation, the current results were not influenced by varying overall soccer conditioning across groups (Table 1). Age distribution was consistent across groups (Table 1) and body mass did not change significantly in any of the groups (Table 4). The moderate group sample sizes may mask possible significant outcomes. However, based on the trivial to moderate effect magnitudes, our findings do not support a recommendation to perform the present training regimes under otherwise identical conditions. Despite the absence of significant differences in the experimental training interventions, the present study may outline directions for future related studies.

### Effort matched sprint training

The two training sessions used were equally rated in terms of session RPE (Table 2). Furthermore, recovery status after two days was not different for the maximal and sub-maximal training groups. Based on these observations, we find it reasonable to conclude that the two repeated-sprint training sessions were effort matched. Blood lactate values obtained after repeated sprints at 90% intensity were below what has been considered “lactate threshold intensity” (2.5–4.0 mmol·L^-1^) in endurance training [23]. In contrast, repeated sprinting at maximal intensity was accompanied with BLa^-^ at or above the typical lactate threshold range described for endurance athletes. Even though BLa^-^ values obtained from sprint and endurance training are not directly comparable, the present data suggest metabolic pathway partitioning differences between 90% and maximal sprinting. Small but meaningful differences in muscle recruitment cannot be excluded as contributory to the observed increase in lactate accumulation at maximal sprinting.

### Effects of training at maximal and sub-maximal intensity

The present results revealed only trivial and non-significant changes in soccer-related sprinting from pre- to post-training for 100UNSUP (Table 3, Fig. 2). Previously, Tønnessen et al. [24] observed unaltered sprint velocity over 0–20 m sprint and improved velocity over 20–40 m as a result of weekly repeated 40-m sprints at maximal or near maximal intensity. This suggests that players are more disposed to adaptations over somewhat longer but less soccer-specific sprint distances. Soccer players perform a high number of brief accelerations during training and games [14]. Thus, one could argue that most players have likely maximized their 0–20 m sprint (acceleration) potential during regular soccer conditioning. While sprint performance remained unchanged in 100UNSUP (Table 3), SL and SR changed significantly from pre- to post-training (Table 4). These changes were greater than the observed typical variation. Our findings are somewhat in contrast to previously published studies stating that individual achievement of sprint velocity corresponds to an optimal self-selected step length/step rate ratio [25] and that a different ratio will produce a lower velocity, so-called negative interaction [26]. In the present study, maximal sprint training induced a significant shift in the step length/frequency relationship “selected” by the athletes, with step frequency increasing 3% from 4.33 to 4.46 steps^.^s^-1^ and step length declining correspondingly. These changes cannot be ascribed to supervision as this group did not receive sprint technique feedback or instruction. Moreover, 100UNSUP demonstrated an increase in BLa^-^ after 15x20 m maximal sprinting in the post-training test, suggesting possible changes in anaerobic energy release, buffering characteristics or muscle recruitment pattern. However, these possible physiological changes were not accompanied with enhanced performance.

Based on both current and previous findings [13], it cannot be concluded that weekly training at 90% velocity is a sufficient sprinting intensity for stimulating adaptation over short sprint distances. Blood lactate and peak heart rate values observed in the present junior soccer players indicate relatively low metabolic stress (Table 2). It is possible that sub-maximal sprint training is more appropriate for typical competitive athletics sprinting distances (100–200 m) compared to 0–20 m accelerations. 20-m sprints are comprised of high to maximal acceleration from a resting state and continuing through the timed distance. In this condition, energy demands during the acceleration phase greatly exceed those at peak velocity [17]. The change in kinetic energy (½mv^2^) is proportional to the square of the change in velocity, such that the 90% sprint condition is associated with a nearly 20% reduction in kinetic energy change (and presumably, muscular energetic demand) compared to maximal sprinting velocity. Due to this non-linearity, a 5% reduction in short sprint velocity during repeated-sprint training over short distances would correspond to 90% workloads in strength training and endurance training and might give a more optimal balance of stress, injury risk reduction and adaptive signal retention. This possibility remains to be explored.

### Effects of supervised training

The present study revealed no significant training effects when supervised and unsupervised sprint training at 90% sprint velocity were compared (Table 3 and Fig. 2). However, the 90SUP group improved Yo-Yo IR1 performance by a moderate margin compared to the other groups. Since Haugen et al. [13] reported unchanged VO_2 max_ after seven weeks of repeated-sprint training at 90% intensity, it is reasonable to assume that locomotion efficiency during high-intensity running has improved in 90SUP. The lack of effects on maximal and repeated-sprint performance may have been affected by the possibility that sprint training at 90% sprint speed is below the lowest effective sprinting intensity for stimulating adaptation. Future studies should therefore explore the effect of supervised training with a gradual increase in intensity from sub-maximal to maximal sprint velocity. Another argument for such a gradual increase in velocity also becomes relevant if one assumes that the athletes gradually enhance sprint performance over the training period. We chose not to control and adjust for possible sprint performance enhancement in 90SUP and 90UNSUP throughout the present intervention period to avoid a mix of different treatment conditions.

Theoretically, the lack of effects with supervised sprint training may be due to poor coaching quality such that the athletes were not able to translate the instructions into practice. However, both training experts used in the present study had many years of experience coaching athletics performers on both national and international levels. Mazzetti et al. [7] and Coutts et al. [8] showed that the presence of a training expert was beneficial for maximal strength and power development over time. In contrast to the present study, the training experts in those studies were allowed to adjust the total training load during the interventions. Based on these observations, one could argue that the effect of expert supervision during training is optimized when combined with greater flexibility in the day-to-day training prescription.

### Training-related constraints

Common challenges in applied studies of this nature are related to constraints with overall team conditioning [1,13,27]. Current analyses confirm these constraints, even though the study was conducted early pre-season where total training load is typically reduced. In CON, we observed ±0.04 s absolute individual variation in mean sprint time between pre- and post-training tests. More importantly, weekly changes in group mean values up to 0.05 s (nearly 2%) were observed in 100UNSUP (Fig. 3), despite consistent frequency and volume of games and training sessions during the intervention period (Table 1). This weekly or seasonal variation is considerably higher than the observed typical variability. The present findings emphasize the need for more detailed information about overall conditioning load, accepting that soccer-specific movements (i.e. brief accelerations, high sprinting velocities or changes of directions) are impossible to assess accurately in groups of players with current technology [28,29]. In principle, the present study could have been accomplished in a more controlled experimental environment, omitting the concurrent soccer training. However, such an approach severely limits the external validity. If improvement of sprinting performance is the primary goal for certain players, future studies should explore the effects of more frequent sprint-training sessions and longer intervention periods, perhaps in combination with other training forms. Future studies should also explore whether it is more effective to structure the players’ weekly soccer training rather than introducing an additional physical conditioning regime. A theoretically perfect conditioning program for certain capabilities may limit other important qualities and vice versa. Coaches and conditioning experts have to balance their training methods and exercises in order to optimize different skills in relation to their contribution to overall soccer performance. Based on the varying individual responses to each of the performed treatments (Fig. 2) and the absence of evidence supporting the choice of specific training methods at the group level, it is essential to diagnose each individual and develop training interventions that target their key physiological and technical weaknesses.
